# Supplementary material for: GM1 Oligosaccharide Efficacy in Parkinson’s Disease: Protection against MPTP
Source: Biomedicines. 2023 Apr 28;11(5):1305. doi: 10.3390/biomedicines11051305 (PMC10216367; doi:10.3390/biomedicines11051305)
Supplement: Supplementary file 1 [file biomedicines-11-01305-s001.zip › biomedicines-2357977-supplementary.pdf]

biomedicines

Supplementary Materials

GM1 Oligosaccharide Efficacy in Parkinson's Disease: Protection against MPTP

Maria Fazzari [1,†], Giulia Lunghi [1,†], Alexandre Henriques [2], Noëlle Callizot [2], Maria Grazia Ciampa [1], Laura Mauri [1], Simona Prioni [1], Emma Veronica Carsana [1], Nicoletta Loberto [1], Massimo Aureli [1], Luigi Mari [3], Sandro Sonnino [1], Elena Chiricozzi [1,*] and Erika Di Biase [1,*]

1 Department of Medical Biotechnology and Translational Medicine, University of Milano, 20054 Segrate, MI, Italy; maria.fazzari@unimi.it (M.F.); giulia.lunghi@unimi.it (G.L.); maria.ciampa@unimi.it (M.G.C.); laura.mauri@unimi.it (L.M.); simona.prioni@unimi.it (S.P.); emma.carsana@unimi.it (E.V.C.); nicoletta.loberto@unimi.it (N.L.); massimo.aureli@unimi.it (M.A.); sandro.sonnino@unimi.it (S.S.)
2 Neuro-Sys, 410 Chemin Départemental 60, 13120 Gardanne, France; alexandre.henriques@neuro-sys.com (A.H.); noelle.callizot@neuro-sys.com (N.C.)
3 Department of Immunology, St. Jude Children's Research Hospital, Memphis, TN 38105, USA; luigi.mari@stjude.org (L.M.)
* Correspondence: elena.chiricozzi@unimi.it (E.C.); edibiase1@mclean.harvard.edu (E.D.B.)
† These authors equally contribute to this work.

S1. Methods

S1.1. MPP+ Dose Effect on CGN

S1.1.1. MTT Assay

Viability of CGN after MPP+ treatments (25, 50, and 100 µM) was determined by MTT assay, as previously reported [38]. Briefly, after 1 h from GM1-OS incubation (100 µM), cells plated in a 96-well were washed and incubated with 100 µL of 2.4 mM MTT (4 mg/mL in RH) for 1 h at 37 °C in a humidified atmosphere of 95 % air/5 % CO_2. Subsequently MTT was carefully removed and replaced with 2-propanol/formic acid, 95/5 (v/v). Plates were gently shacked prior to read the absorbance at 570 nm with a microplate spectrophotometer (Wallac 1420 VICTOR2TM, Perkin Elmer).

S1.1.2 Morphological Analysis for Neurite Outgrowth Evaluation

After MPP+ challenge in the presence or absence of GM1-OS, CGN, were observed by phase contrast microscopy (20X objective, Olympus BX50 microscope; Olympus, Tokyo, Japan). At least 10 fields from each well were photographed for each experiment.

S2. Supplementary Figures

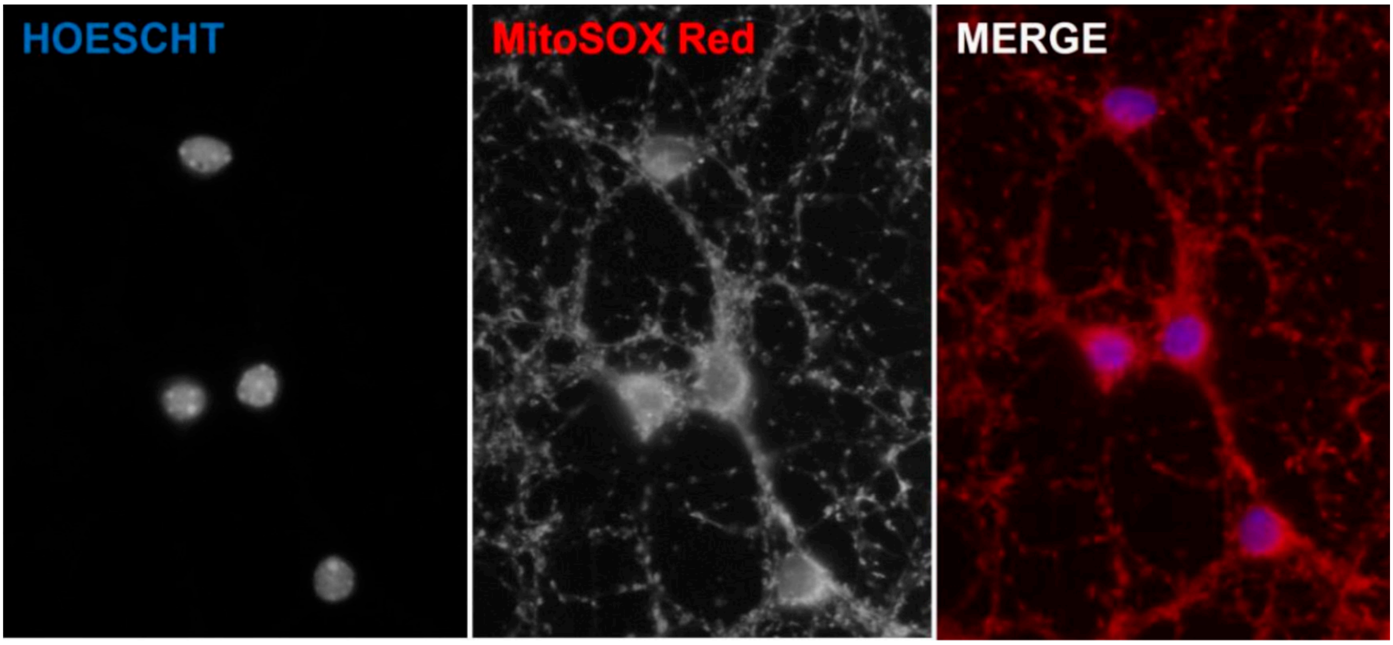

Figure S1. Identification of $O_2^{\bullet -}$ content in mitochondria by MitoSOX™ red dye. Representative image of day 14 primary CGNs [47] stained with 1 μM MitoSOX red dye in HBSS with Ca^{2+} and Mg^{2+} for 10 min at 37 °C. After MitoSOX incubation, PBS-washed CGNs were fixed and nuclei were stained by Hoechst dye. Images were acquired using a NikonEclipse Ni upright microscope with 100X objective (Red: Mitosox; Blue: Nuclei).

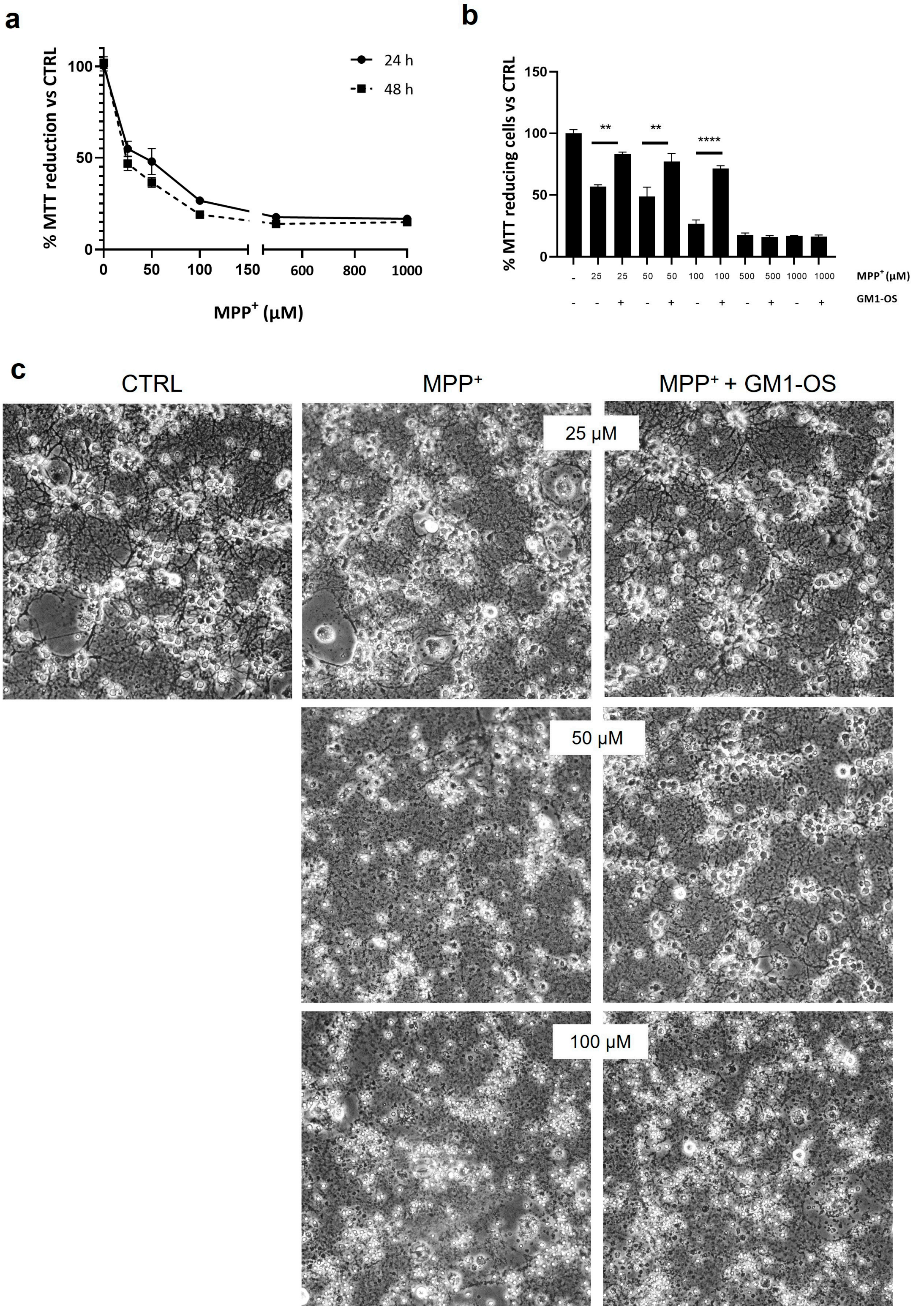

Figure S2. GM1-OS protected CGN injured with MPP^+. (**a**) Dose-response effect of MPP^+ exposure on CGN after 24 h (straight line) or 48 h (dashed line). MTT assay was performed to measure cell viability; (**b**) MTT assay of MPP+ exposed CGN at the indicated concentrations in the presence or absence of 100 µM GM1-OS administered 1 h prior to MPP^+; (**c**) Representative phase contrast images of CGN exposed to MPP^+ at the indicated concentrations in the presence or absence of 100 µM GM1-OS (administered 1 h prior to MPP^+). Magnification 20X. All values are expressed as mean ± SEM (n = 4. ** $p < 0.01$, **** $p < 0.0001$ One-way ANOVA followed by Tukey's multiple comparisons test).
